# In Reply to: Comments on Behavior of Healthcare Workers After Injuries From Sharp Instruments

**DOI:** 10.5812/traumamon.17077

**Published:** 2014-01-25

**Authors:** Mohsen Adib-Hajbaghery, Mohammad Sajjad Lotfi

**Affiliations:** 1 Department of Medical-Surgical Nursing, Kashan University of Medical Sciences, Kashan, IR Iran

**Keywords:** Prevention and Control, Needlestick Injuries, Health Personnel

*Dear Editor,*

After the publication of our previous study (1), Alavian submitted a letter which emphasized the importance of postinjury follow-ups (2). Consistent with our previous reports, Alavian has noticed that health care workers are at higher risk of hepatitis B virus (HBV) and hepatitis C virus (HCV) infections, through the contact with blood and infected fluids following injuries caused by sharp instruments. In addition to our finding, around 90% of cases enrolled in the study had received hepatitis B vaccination; Alavian enquired why the authors did not present any data regarding the post-vaccination titer of anti-HBs antibody of the enrolled subjects. Though it is important to determine the post-vaccination anti-HBs antibody level (as an important determinant of transmission risk), we did not address this issue in our study and it may be considered as a limitation of our study. Alavian has also referred to another finding in our study that 38.3% of the enrolled subjects had a history of injury from needles and sharp instruments in the last six months. Then, he mentioned higher rates of sharp instrument injuries reported in the literature. Although this is true, the rates of sharp instrument injuries vary among healthcare workers in different countries. For instance, the incidence of sharp instrument injuries varies from 31.4% in Germany to 79% in India (1, 3, 4). Such differences may be related to many factors such as the knowledge and approaches of healthcare works toward this issue, their workloads, and also the performance of the supportive and supervisory systems in establishing and executing the protocols used for prevention of these dangerous injuries. Such differences may also be observed in different healthcare settings. For example in a study by Shokuhi et al. the highest prevalence of sharp instrument injuries was reported among medical residents (5), while in a report published by the world health organization, the highest number of injury per healthcare worker each year was among nurses (6). However, sharp instrument injury reports are underestimated, because of the different reasons such as recall bias, response rate and low rate of reporting (1). We agree with Alavian that it is necessary to educate healthcare workers especially nurses and midwives, because most sharp instrument injuries occurred among this group. However, it seems that some regulatory mandates are needed to be implemented for the prevention of sharp instrument injuries, because as the recent study showed, nurses do not follow the precautions for preventing needlestick injuries unless such regulatory rules and regulations are implemented (7). 
